# Host bone marrow-derived IL-12 enhances donor T cell engraftment in a mouse model of bone marrow transplantation

**DOI:** 10.1186/1756-8722-7-16

**Published:** 2014-02-28

**Authors:** Katarzyna A Darlak, Ying Wang, Jian-Ming Li, Wayne AC Harris, Cynthia R Giver, Chunzi Huang, Edmund K Waller

**Affiliations:** 1 Department of Hematology and Medical Oncology, Winship Cancer Institute, Emory University, 1365B Clifton Rd. NE, Room B5119, Atlanta, GA, USA; 2 Leukemia Center, Institute of Hematology and blood Diseases Hospital, Chinese Academy of Medical Sciences, Peking Union Medical College, Tianjin, China

**Keywords:** Interleukin-12, Engraftment, Transplantation, T-cells

## Abstract

**Background:**

Donor cell engraftment is critical for the success of allogeneic bone marrow transplants. Graft failure is a result of donor cells either failing to engraft initially or being eliminated at later time points. Donor cell engraftment is facilitated by donor T cells, which eliminate residual host hemato-lymphoid effector cells such as NK cells and T cells.

**Methods:**

We aimed to explore the role of host hematopoietic cell derived IL-12 on donor cell engraftment in a murine model of BMT. We established radiation chimeras by transplanting C57BL6/J (B6) mice with BM from either congenic B6 mice or IL-12p40 KO mice. These WT → WT or IL-12 KO → WT chimeras then underwent a secondary transplant with allogeneic (FVB) BM. Survival, engraftment, donor T cell expansion, cytokine production by donor T cells, as well as expression of stimulatory markers on donor T cells was analyzed.

**Results:**

Mice whose residual host hematopoietic cells were capable of producing IL-12 had modestly higher survival, higher donor T cell engraftment, and significantly higher donor erythroid engraftment. We have also found that an increased number of donor T cells in IL-12 KO → WT chimeras have a regulatory phenotype, expressing FoxP3, producing lower levels of TNF-α, higher levels of IL-10, and expressing higher levels of ICOS as well as PD-1 on CD4+ T cells.

**Conclusions:**

To our knowledge, this is the first report of a beneficial role of IL-12 production by host cells in the context of bone marrow engraftment in a murine model of BMT. These findings support the clinical use of exogenous IL-12 for use in settings where graft failure is of concern.

## Background

Donor hematopoietic cell engraftment is the cornerstone of all successful allogeneic bone marrow transplantation (BMT). Allograft rejection occurs when donor cells fail to engraft initially, or when there is a loss of donor cells at a later time point following initial engraftment. While the overall frequency of graft failure in BMT is less than 5%, graft failure is still a major concern when the source of the allograft is a T cell depleted (TCD) human leukocyte antigen (HLA) – haploidentical donor, in cord blood transplants [1], in BM grafts from unrelated donors [2], or in patients where non-myeloablative conditioning is used. LeBlanc et al. examined patients with various hematological malignancies or solid tumors that had received non-myeloablative conditioning or a higher-intensity conditioning regimen followed by a hematopoietic stem cell transplant (HSCT) from either HLA-identical siblings or unrelated donors [3]. It was found that 6/24 patients receiving non-myeloablative conditioning experienced graft failure, compared with 1/34 patients in the higher intensity conditioned group [3].

The addition of donor T cells to the BM graft has been shown to facilitate engraftment in animal models [4,5]. The current model for hematopoietic cell engraftment in allogeneic BMT is that host dendritic cells (DC) activate donor T cells, which then promote engraftment by eliminating radio-resistant cytotoxic host immune cells, especially natural killer (NK) cells and host T-cells. Host DC have also been shown to initiate graft-versus-host disease (GvHD), and inactivation of host DC can prevent GvHD [6]. The interplay between residual host DC and T cells represents a delicate balance between engraftment and GvHD.

We have recently shown that tumor-bearing mice receiving BM grafts which were depleted of myeloid DC (mDC) had enhanced survival and donor T-cell expansion compared with mice receiving unmanipulated BM grafts, which contained both mDC and plasmacytoid DC (pDC) [7]. Using interleukin-12 knockout (IL-12p40 KO) mice as BM donors, it was found that the increased survival in mice receiving mDC depleted grafts was dependent on the production of IL-12p40 by donor pDC [7]. IL-12 is produced by DCs that can drive the development of donor type 1 helper T-cells (T_H_1) and type 1 cytotoxic T-cells (Tc1). It has also been shown that administration of exogenous IL-12 in a mouse model of BMT can reduce GvHD while preserving the graft-versus-leukemia effect (GvL) [8].

While the role of IL-12 has been extensively studied in the context of GvL and GvHD, the role of IL-12 in graft failure has yet to be fully examined. Administration of exogenous IL-12 can protect the bone marrow from the effects of lethal irradiation, at the expense of sensitizing the intestinal tract [9]. Administration of exogenous IL-12 at certain doses has also been shown to facilitate hematopoietic engraftment following lethal irradiation [10]. We aimed to explore the role of IL-12 produced by residual host hematopoietic cells in donor cell engraftment. As IL-12 can be produced by non-hematopoietic cells, including keratinocytes, osteoblasts, epithelial cells, and endothelial cells, we established radiation chimeras in C57BL6/J (B6) mice in which only hematopoietic cells lacked the ability to produce IL-12p40 [11,12]. After confirmation of donor hematopoietic chimerism, mice received lethal irradiation followed by an allogeneic BMT (FVB or B10.BR → B6 model). Survival, engraftment, donor T cell expansion (using luciferase positive T-cells), and cytokine production were examined. It was found that murine recipients that lacked production of IL-12p40 by host hematopoietic cells had lower survival, a lower level of donor hematopoietic engraftment, a reduced percentage T_H_1 cytokine producing donor T cells, and an increased percentage of regulatory T cells in the spleen. This is the first report that host-derived IL-12 plays a significant role in engraftment and has implications for the use of IL-12 in transplant settings where graft failure is of concern.

## Results

### Host-hematopoietic-derived IL-12 enhances survival after BMT

**Figure 1:**
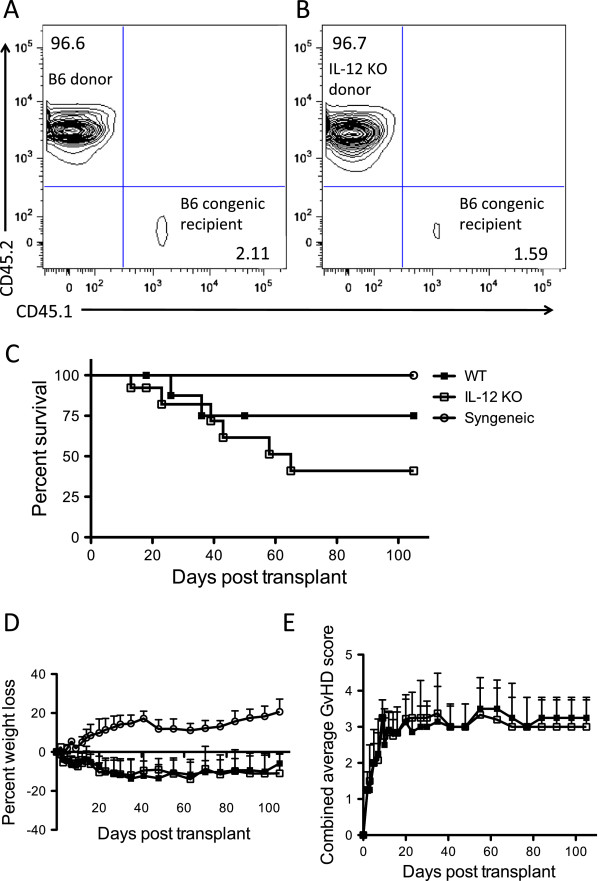
**Host-derived IL-12 enhances survival following BMT Radiation chimeras were established by lethally irradiating (11 Gy) B6 Pepboy (C57BL6/J congenic, CD45.1) mice followed by transplant with 5 × 10**^**6 **^**BM Cells from either B6 (or BA – B6 congenic) or IL-12-p40 KO (B6 background) mice.** Chimerism was confirmed a minimum of 50 days post-transplant by flow-cytometric analysis of congenic markers on total nucleated cells (CD45.1, CD45.2) for both B6 **(A)** and IL-12p40 KO **(B)** chimeras. Flow plots are data from one representative mouse per group. Chimeric mice were then conditioned with 9 Gy irradiation and transplanted with 5 × 10^6^ FVB TCD BM cells along with 3 × 10^5^ MACS-purified *luc +* FVB T-cells. A syngeneic transplant was also performed using non-radiation chimera FVB mice as recipients. Survival **(C)**, percent weight loss from initial starting weight **(D)**, and combined GvHD scores **(E)** were monitored after transplant. Data shown is combined from 3 independent experiments of 4–5 mice per group (WT and IL-12p40 KO) or 3 mice per group (syngeneic).

In order to examine the role of host-immune cell derived IL-12, radiation chimeras were established in order to create mice that lacked IL-12 in the hematopoietic compartment only. One day prior to transplant, congenic B6 mice were lethally irradiated with 11 Gy total body irradiation (TBI) and transplanted i.v. with 5 × 10^6^ BM cells from B6 or IL-12p40 KO mice. A minimum of 50 days after transplant, donor chimerism of total nucleated cells in the blood of both B6 and IL-12 KO radiation chimeras was confirmed by flow cytometric analysis using congenic markers (CD45.1, CD45.2, CD90.1, CD90.2) (Figure 1A,B, representative data). Chimerism was found to be greater than 95% in all radiation chimeras.

Radiation chimeras underwent a secondary transplant following irradiation with 9 Gy TBI one day prior to transplant. In B6 radiation chimeras, radioresistant host-hematopoietic cells would be capable of producing IL-12 following irradiation and transplant, along with donor hematopoietic cells. In IL-12 KO radiation chimeras only the donor-derived hematopoietic cells would produce IL-12, as residual host hematopoietic cells were of IL-12 KO origin.

One day after the second irradiation course, chimeras were transplanted i.v. with 5 × 10^6^ TCD BM cells from FVB donors along with 3 × 10^5^ luciferase positive (*luc+*) FVB T-cells. Control transplant mice (syngeneic) were FVB mice transplanted i.v. with 5 × 10^6^ TCD BM cells from FVB donors along with 3 × 10^5^*luc +* FVB T-cells. Survival of mice was monitored daily. Weight loss and clinical GvHD scores were monitored twice weekly after transplant, as described by Cooke et al. [13]. IL-12 KO → WT mice had a median survival of 65 days post-transplant (41% survival at day 105 post-transplant), which was lower compared with WT → WT mice (median survival day undefined, 75% survival at day 105 post-transplant), though not significant (p = 0.24) (Figure 1C). All syngeneic-transplanted mice survived to day 105. Percent weight loss from initial starting weight and GvHD scores were similar between WT → WT and IL-12 KO → WT radiation chimeras (Figure 1D,E,F). Control transplanted mice did not experience weight loss after transplant (Figure 1D).

### Host-hematopoietic-derived IL-12 enhances donor T-cell engraftment after BMT

**Figure 2:**
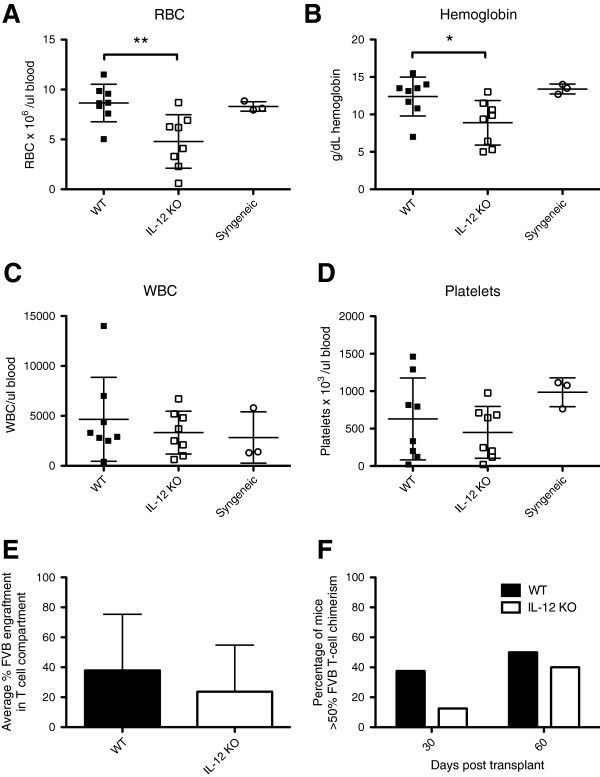
**Host-derived IL-12 enhances erythroid and T-cell engraftment 30 days post-transplant.** Radiation chimeras (B6 or BA and IL-12p40 KO) were conditioned with 9 Gy irradiation and transplanted with 5 × 10^6^ FVB TCD BM cells along with 3 × 10^5^ MACS-purified *luc +* FVB T-cells. A syngeneic transplant was also performed using non-radiation chimera FVB mice as recipients. The levels of red blood cells (RBC) **(A)**, hemoglobin **(B)**, white blood cells (WBC) **(C)**, and platelets **(D)** were measured in the blood 30 days post-transplant. The percentage of cells of donor–origin (FVB) in the T-cell compartment on day 30 post-transplant was determined by flow-cytometric analysis of congenic markers (CD45.1, CD45.2, CD90.1, CD90.2) after gating on CD3+ cells **(E)**. The percentage of mice in each group (WT or IL-12p40 KO) that exhibited greater than 50% FVB T-cell chimerism are shown in **(F)**. **, *p* < 0.01 comparing RBC of WT vs. IL-12p40 KO at day 30; *, *p* < 0.05 comparing Hemoglobin of WT vs. IL-12p40 KO at day 30. Data shown is combined from 2 independent experiments of 4 mice per group (WT and IL-12p40 KO) or 3 mice per group (syngeneic).

Next we determined the effect of host hematopoietic derived IL-12 on the engraftment of leukocytes, red blood cells, and platelets. On day 30 post-transplant, we measured the red blood cell (RBC) count, white blood cell (WBC) count, platelet number, and hemoglobin levels in the blood of recipient mice. Recipient mice in which host immune cells were capable of producing IL-12 had significantly higher erythroid engraftment as seen by significantly higher RBC counts and hemoglobin levels (Figure 2A,B respectively). WBC counts in the blood of recipients previously engrafted with WT BM were slightly higher, though not significant (Figure 2C). Platelet counts were not significantly different among groups (Figure 2D). We also measured the percentage of T cells of donor (FVB) origin as a percentage of total T cells. Radiation chimeras previously engrafted with WT BM had a higher percentage of donor T cells (37.87 ± 13.25) on day 30 post-transplant compared with IL-12 KO → WT chimeras (23.69 ± 10.98) (Figure 2E). Standard deviation in Figure 2E is very high in both groups, as most mice had engrafted primarily with FVB (80% or higher donor T cells of FVB origin), or had failed to engraft (lower than 40% donor T cells of FVB origin). On day 30 post-transplant, 40% of WT → WT chimeras had greater than 50% donor T cell engraftment, while only 10% of IL-12 KO → WT chimeras had greater than 50% donor T cell engraftment (Figure 2F). Mice that had failed to engraft died. Among surviving mice on day 60 post-transplant, 50% of WT → WT chimeras had greater than 50% donor T cell engraftment, compared with 40% of IL-12 KO → WT chimeras (Figure 2F).

**Figure 3:**
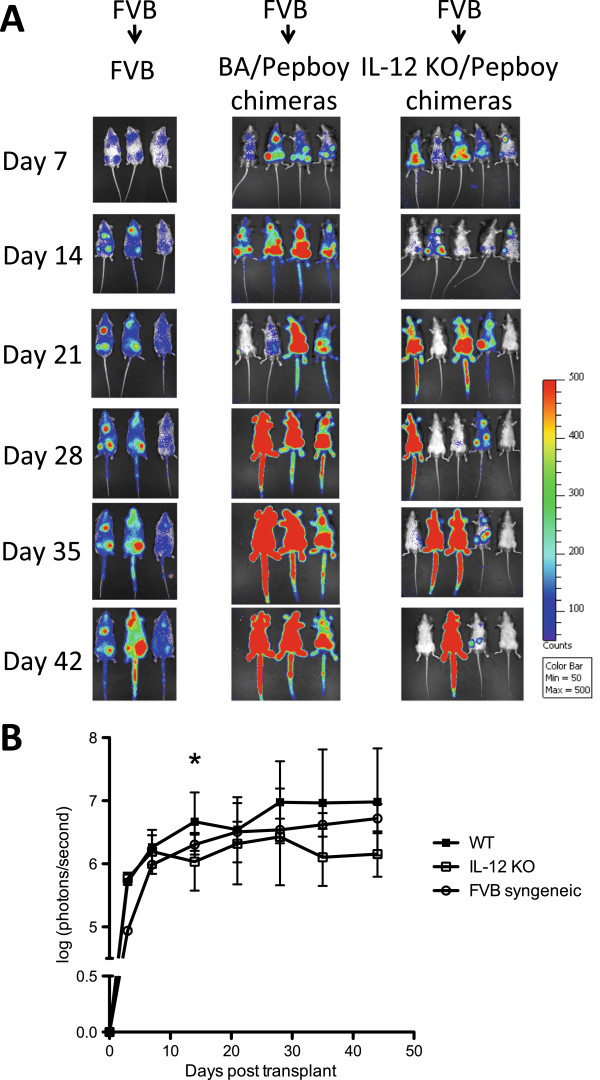
**Donor T cell engraftment is increased and occurs more rapidly in mice producing host immune-derived IL-12.** Radiation chimeras (B6 or BA and IL-12p40 KO) were conditioned with 9 Gy irradiation and transplanted with 5 × 10^6^ FVB TCD BM cells along with 3 × 10^5^ MACS-purified *luc +* FVB T-cells. A syngeneic transplant was also performed using non-radiation chimera FVB mice as recipients. **(A)** In-vivo bioluminescent images of mice were taken weekly beginning 7 days post transplant and ending 42 days post transplant. The color bar represents the scale used for the pseudocolor image. Images are representative from one of two independent experiments of 4–5 mice per group (3 mice per group, syngeneic). **(B)** Total signal in the region of interest (ROI) (photons/sec) was determined for the whole-body on days 7, 13, 21, 28, 35, and 42 post-transplant. ROI data has been log transformed, however statistical analyses were completed on raw data. Data are combined from two independent experiments of 4–5 mice per group (3 mice per group, syngeneic); *, *p* < 0.05 comparing WT vs. IL-12p40 KO on day 14 post-transplant; p = 0.092 and p = 0.098 comparing WT vs. IL-12p40 KO on days 28 and 35 post transplant, respectively.

Since the radiation chimeras were transplanted with FVB TCD BM and luciferase-positive FVB T-cells, donor T-cell engraftment could be tracked using *in vivo* bioluminescent imaging at multiple time points post-transplant. Mice were imaged every week beginning at 7 days post-transplant and continuing until 42 days post-transplant. Representative imaging data from one experiment is shown in Figure 3A. A greater percentage of the WT → WT radiation chimeras had donor T cell bioluminescent signals, and the signals were of greater intensity, than that of IL-12 KO → WT radiation chimeras (Figure 3A). At day 42, only one IL-12 KO → WT radiation chimera had a strong bioluminescent signal, and the signal from donor T cells in 2/4 IL-12 KO → WT chimeras was not detectable. The strength of the signal from each mouse in photons/second was quantified. WT → WT chimeras had a higher average signal overall compared with IL-12 KO → WT chimeras (Figure 3B). The signal from WT → WT radiation chimeras was significantly higher on D14 post transplant (p < 0.05), as well as slightly higher on days 28 and 35 post-transplant (p = 0.092 and p = 0.098, respectively) (Figure 3B). T cells in WT → WT mice had a bioluminescent signal strength similar to or even higher than that of FVB mice receiving a syngeneic transplant.

### The absence of host-hematopoietic-derived IL-12 drives donor CD4+ T-cells towards a tolerogenic phenotype

**Figure 4:**
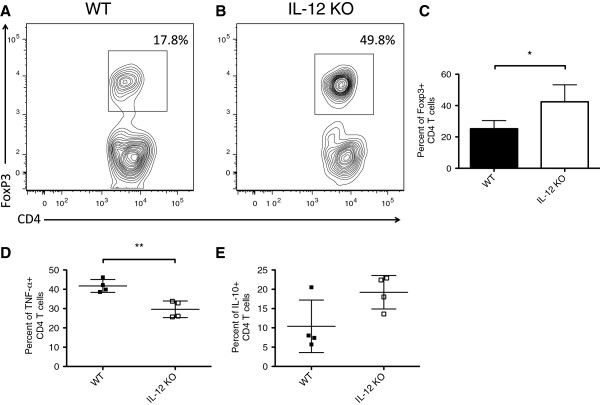
**Host immune-derived IL-12 drives donor-T cells towards a Th1 phenotype.** Radiation chimeras (B6 or BA and IL-12p40 KO) were conditioned with 9 Gy irradiation and transplanted with 5 × 10^6^ BA.B10 TCD BM cells and 3 × 10^5^ MACS-purified B10.BR T-cells. Recipient splenocytes were prepared 10 days post transplant and analyzed via flow-cytometry for the percentages of regulatory T cells as well as TNF-α and IL-10 producing donor T-cells. Donor-T cells were gated on using congenic markers (CD45.1, CD45.2, CD90.1) and CD3. Regulatory T cells were defined as T cells expressing both CD4 and FoxP3. Representative flow plots for B6 **(A)** and IL-12p40 KO **(B)** radiation chimeras are shown. Percentages of FoxP3+ T cells in the CD4 T cell compartment are shown in **(C)**; *, *p* < 0.05 comparing WT vs. IL-12p40 KO. Data shown are from two independent experiments of 4–5 mice per group. Percentages TNF-α and IL-10 producing CD4+ donor-T cells are shown in **(D)** and **(E)**, respectively. IL-10 data are representative from one of two independent experiments with 4–5 mice per group using pre-transplant irradiation of 9 Gy or 10 Gy (9 Gy is shown). TNF-α data is combined from two independent experiments with 4–5 mice per group. **, *p* < 0.01 comparing WT vs. IL-12p40 KO.

While survival and donor-T cell engraftment was higher among WT radiation chimeras compared with IL-12 KO radiation chimeras, we next aimed to determine whether the phenotype of engrafted T cells was different between groups. On day 10 post-transplant, percentages of T cells expressing FoxP3, producing Tumor necrosis factor-α (TNF-α) or interleukin-10 (IL-10), and expressing induced costimulator (ICOS) or Programmed Death-1 (PD-1) were determined. IL-12 KO → WT chimeras had a higher percentage of donor CD4 T cells expressing the regulatory T cell marker FoxP3, as determined by intracellular staining for FoxP3 (p < 0.05, Figure 4C). Representative flow plots for WT → WT and IL-12 KO → WT radiation chimeras are shown in Figure 4A,B, respectively. The ability of host hematopoietic cells to secrete IL-12p40 also had an impact on the percentage of donor CD4 T cells that produce IL-10 and TNF-α. A greater percentage of donor CD4+ T cells from WT chimeras produced TNF-α compared with IL-12 KO chimeras (p < 0.01, Figure 4D), while a slightly higher percentage of CD4+ T cells from IL-12 KO chimeras produced IL-10 compared with WT chimeras (p = ns, Figure 4E).

**Figure 5:**
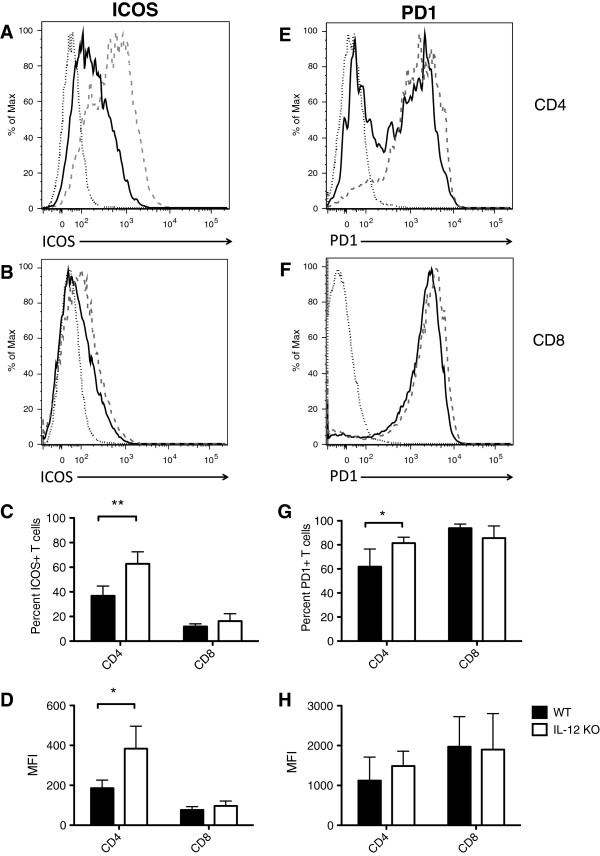
**Host immune-derived IL-12 limits ICOS and PD1 expression by CD4+ donor T cells.** Radiation chimeras (B6 or BA and IL-12p40 KO) were conditioned with 9 Gy irradiation and transplanted with 5 × 10^6^ BA.B10 TCD BM cells and 3 × 10^5^ MACS-purified B10.BR T-cells. Recipient splenocytes were harvested 10 days post transplant and analyzed via flow-cytometry. Donor T cells were gated on using congenic markers (CD45.1, CD45.2, CD90.1) as well as CD3, and were then further differentiated using CD4 and CD8. Representative histograms are shown for ICOS expression in CD4+ **(A)** and CD8+ **(B)** donor T-cells and PD1 expression in CD4+ **(E)** and CD8 + **(F)** donor T-cells (Isotype – fine dotted line, WT – black solid line, IL-12p40 KO – gray dashed line). ICOS and PD-1 expression by CD4+ and CD8+ donor T cells was analyzed; Percentages of donor T-cells expression ICOS **(C)** and PD1 **(G)** are shown as well as the median fluorescence intensity (MFI) of the signal for ICOS **(D)** and PD1 **(H)**. **, *p* < 0.01 comparing percentages of ICOS + CD4+ donor T-cells in WT vs. IL-12p40 KO; *, *p* < 0.05 comparing MFI of ICOS signal in CD4+ donor T-cells in WT vs. IL-12p40 KO; *, *p* < 0.05 comparing percentages of PD1+ CD4+ donor T-cells in WT vs. IL-12p40 KO. Data shown for ICOS are representative from one of two independent experiments with 4 mice per group using pre-transplant irradiation of 9 Gy or 10 Gy (9 Gy is shown). Data shown for PD1 are combined from two independent experiments of 4 mice per group.

Signaling through ICOS has been shown to contribute to BM graft rejection and GvHD [14]. We measured ICOS expression in WT and IL-12 KO chimeras on donor CD4 and CD8 T cells 10 days post-transplant. IL-12 KO chimeras (gray dashed line) had higher expression of ICOS on CD4+, but not CD8+ T cells compared with WT chimeras (black solid line) (representative histograms, Figure 5A – CD4, 5B – CD8). Isotype control staining is indicated by a gray dotted line. The percentage of CD4+ T cells expressing ICOS was higher in IL-12 KO chimeras (p < 0.01, Figure 5C), as well as the median fluorescence intensity of the signal (p < 0.05, Figure 5D), compared with WT chimeras.

The PD-1/Programmed Death Ligand 1 (PDL1) pathway is critical for inducing peripheral deletional tolerance of anti-donor CD8 T cells in BMT [15]. We analyzed the expression of PD-1 on CD4+ and CD8+ T-cells on day 10 post-transplant. Surprisingly, the level of PD-1 on the surface of CD8+ T cells was similar between WT (solid black line) and IL-12 KO chimeras (gray dashed line) (Isotype control, gray dotted line) (Figure 5F). CD4+ T cells in IL-12 KO → WT chimeras, however, had higher PD-1 expression (Figure 5E). The percentage of CD4+ T cells expressing PD-1 was significantly higher in IL-12 KO → WT chimeras (p < 0.05, Figure 5G), and the MFI was slightly higher (Figure 5H), compared with WT → WT chimeras. The percentages of CD8 T cells expressing PD-1 and the MFI of the PD-1 signal were not different comparing donor cells in WT → WT vs. IL-12 KO → WT chimeras (Figure 5G,H).

## Discussion

The role of host-derived IL-12 in engraftment has not been examined previously. IL-12 promotes the maintenance of T-helper-type 1 (T_H_1) cells that are necessary for elimination of residual host hematopoietic cells in the process of engraftment. While increased T_H_1 polarization might be thought to increase GvHD, administration of exogenous IL-12 has also previously been shown to prevent GvHD while preserving GvL effects [8,16-18]. Proper engraftment is a paramount concern in clinical settings such as when the donor is HLA-haploidentical to the recipient, in cord blood transplants, and in patients where non-myeloablative conditioning is used. Engraftment is also a challenge in treatment of sickle cell anemia with HSCT, due to prior host immunity resulting from numerous blood transfusions in the patient’s history. While IL-12 has been examined as a therapeutic agent in reducing the severity of GvHD, we propose that these findings could be translatable to promoting engraftment in settings where graft failure is of concern.

In order to achieve stable engraftment in the presence of cytotoxic host cells not eliminated by irradiation, it is thought that host DCs directly activate donor T cells, which then eliminate the remaining cytotoxic host cells. Wang et al. showed that host DC can activate donor CD4+ T cells, which in turn activate donor APC [14]. These donor APCs are then capable of cross presenting minor histocompatibility antigens (miHA) in an MHC-matched, miHA-mismatched, model to donor CD8+ T cells [14]. Production of IL-12 by DCs promotes maintenance of T cells in a T_H_1 phenotype after commitment to a T_H_1 lineage by T-bet and subsequent upregulation of the IL-12β2 subunit of the IL-12 receptor on T cells [19,20].

Since IL-12 producing APCs are somewhat resistant to radiation, we hypothesized that the production of IL-12 by residual host APCs would aid in the process of engraftment by promoting T_H_1 immunity while not increasing GvHD severity. Radiation chimeras were established where all cells (host and donor) were capable of producing IL-12 (WT → WT chimeras), as well as chimeras in which only the host hematopoietic cells were incapable of IL-12 production (IL-12 KO → WT chimeras). A dose of 9 Gy was chosen for the second BMT using radiation chimeras as recipients, as this is generally the highest tolerated dose for a second transplant.

Our results show that mice whose host hematopoietic cells were capable of IL-12 production had increased survival without higher levels of GvHD or increased weight loss. Donor T cell engraftment in mice whose residual host-hematopoietic cells lacked the ability to produce IL-12 was reduced compared with WT → WT mice, as seen by both flow cytometric analysis of chimerism, as well as in *in vivo* bioluminescent images of mice engrafted with *luc +* donor T cells (Figure 2E,F, Figure 3). WT → WT chimeras had a higher percentage of donor CD4+ T cells producing TNF-α and a lower percentage of CD4+ T cells producing IL-10 on day 10 following transplant compared IL-12 KO → WT chimeras (Figure 4D,E). This suggests that host derived IL-12 is promoting the maintenance of donor T cells of a T_H_1 phenotype. These host cells can prime donor CD8+ T cells to eliminate cytotoxic host effectors that prevent engraftment.

WT → WT chimeras also had a lower percentage of donor CD4+ T cells expressing the T-reg associated transcription factor FoxP3+ compared with IL-12 KO → WT chimeras (Figure 4A-C). If a large percentage of donor CD4+ T cells are of a regulatory phenotype, they are unable to prime donor CD8+ T cells to eliminate cytotoxic host cells. We also found that ICOS was present in higher levels on CD4+ and CD8+ T cells in mice whose host-hematopoietic cells were incapable of producing IL-12.

ICOS is a CD28 superfamily member whose expression is induced on CD4+ and CD8+ T cells following T cell activation [21]. ICOS on T cells binds ICOS ligand (ICOSL), which is upregulated on APCs activated by TNF-α or TLR triggering by LPS [22,23]. ICOS binding with ICOSL can stimulate IL-10 production by T cells [21]. Taylor and Blazar found that ICOS blockade (via either ICOS KO mice or ICOS monoclonal antibodies) led to higher engraftment rates in mice receiving TCD BM following non-myeloablative irradiation (5.5 Gy). Our data coincides with these findings, as mice whose host-hematopoietic cells did not produce IL-12 had lower engraftment, and higher levels of ICOS on CD4+ and CD8+ T cells (Figure 5A-D).

We also found higher percentages of PD-1+ CD4+ T cells in mice lacking the capacity to produce IL-12 by host immune cells (Figure 5G). PD-1 is also a member of the CD28 superfamily and is expressed by activated T cells [24-26]. PD-1 binds to PDL1, which is expressed on all hematopoietic and many non-hematopoietic cells, and PD Ligand 2 (PDL2), which is found on DCs and macrophages [27-30]. PD-1 binding with ligands leads to negative regulation of activated T cells. The PD-1/PDL1 pathway is known to induce deletional tolerance of alloreactive CD8+ but not CD4+ T cells in BMT [15]. While we did not see a difference in the PD-1 expression of CD8+ T cells, there were differences among CD4+ T cells. We speculate that the higher percentages of PD-1+ CD4+ T cells in IL-12 KO chimeric recipients could lead to generation of DC with enhanced immunosuppressive properties [31,32].

It is important to note that the IL-12 KO mice used in this study are IL-12p40 KO, resulting in recipient mice deficient in both IL-12 and IL-23 in the host-hematopoietic compartment. IL-23 is produced by activated DCs and macrophages upon pathogen recognition receptor activation and can stimulate the proliferation of human and mouse activated and memory T cells, but not naïve T cells *in vitro* as well as induce IFN-γ production by T cells [33]. IL-23 is also distinct from IL-12 in that it promotes the maintenance of T_H_17 cells [34]. IL-23 is also produced by APCs following BMT, subsequently inducing IL-22 expression, which has been shown to limit intestinal and liver GvHD pathology [35]. Interpretation of results must account for the potential role of IL-23 in this system, along with the consideration that while the host is lacking both IL-12 and IL-23, donor hematopoietic cells are capable of producing both cytokines. The absence of IL-12 and IL-23 in the host hematopoietic compartment may lead to reciprocal changes in other cytokine and signaling pathways [36,37]. Notably, we have previously shown that the percentages of donor T-cells producing IL-17 were increased in spleens from recipients transplanted with IL-12p40 KO BM, compared with WT BM, 10 days post-transplant [7].

## Conclusions

Our results demonstrate that IL-12 production by residual host cells following irradiation and transplant is a critical factor contributing to successful engraftment. Mice whose host hemato-lymphoid cells lacked the capacity to produce IL-12 had reduced donor T cell engraftment, lower levels of erythroid engraftment, reduced TNF-α production by donor T cells, higher percentages of FoxP3+ CD4+ T cells, and higher expression of ICOS and PD-1 on CD4+ T cells after transplantation. Administration of exogenous IL-12 has been examined by many groups as an anti-tumor therapy, as well as a therapy for alleviating GvHD, and these results support the potential clinical application of IL-12 as a therapy to promote engraftment.

## Materials and methods

### Mice

FVB/NJ (FVB, H-2K^q^, CD45.1, CD90.1), C57BL6/J (B6, H-2K^b^, CD45.2, CD90.2), B10.BR (H-2K^k^, CD45.2, CD90.2, congenic B6 Pepboy (B6.SJL-*Ptprc*^*a*^*Pep3*^*b*^/BoyJ, H-2K^b^, CD45.1, CD90.2), and IL-12p40 KO (B6.129S1-*Il12b*^*tm1Jm*^/J, H-2K^b^, CD45.2, CD90.2) mice were purchased from The Jackson Laboratory (Bar Harbor, ME). Congenic strains expressing CD90.1 and CD45.2 on a B6 (H-2K^b^) background (BA), and CD90.1, CD45.2 on a B10.BR (H-2K^k^) background (BA.B10) were backcrossed 10 generations to the parental strain and bred at Emory University Animal Care Facility (Atlanta, GA, USA). The *luc +* transgenic mice (FVB-L2G85) on the FVB background were a gift from Dr. Robert Negrin (Stanford University) [38]. All transplant recipients were monitored daily for survival. According to the Institutional Animal Care and Use Committee (IACUC) protocol, moribund mice, mice with greater than 25% weight loss, and mice surviving until the end of the experiment, were euthanized and considered to have died on the day following euthanasia for analysis of post transplant survival. Mice were scored for clinical signs of GvHD by weight loss, posture, activity, fur texture, and skin integrity twice weekly for the first 30 days post transplant, and once weekly thereafter, using a GvHD scoring system described by Cooke et al. [13]. Procedures conformed to the National Institutes of Health (NIH, Bethesda, MD) animal care guidelines and were approved by the Emory University IACUC.

### Cell preparation

Donor mice were killed in a humane manner and femurs, tibias, and spleens were removed ascetically. BM cells and splenocytes were harvested with sterile phosphate buffered saline (PBS). T cells were purified by incubating splenocytes with a cocktail of biotinylated anti-CD11b, anti-B220, anti-DX5, and anti-TER119 antibodies, followed by anti-biotin microbeads, and negative magnetic activated cell sorting (MACS) selection using an LS magnetic column (MACS, Miltenyi Biotec). CD3+ T cells were depleted from BM cells by incubation of BM with biotinylated CD3 antibodies, followed by anti-biotin microbeads, and negative MACS selection using an LS magnetic column (MACS, Miltenyi Biotec).

### Radiation chimeras

On day-1 prior to transplantation, recipient congenic B6 Pepboy mice (CD45.1) received a total of 11 Gy irradiation, divided into two doses of 5.5 Gy given 3 hours apart [39]. On day 0, mice were injected i.v. with 5 million BM cells from WT B6 donors (CD45.2) or IL-12p40 KO donors (CD45.2). Chimerism was confirmed a minimum of 50 days post-transplant using flow cytometric analysis of blood stained with anti-CD3-PE-Cy7, anti-CD19-APC-Cy7, anti-CD45.1-PE, anti-CD45.2-FITC, anti-CD90.1-PerCP, and anti-CD90.2-APC (BD Biosciences, San Diego, CA).

### Secondary transplant

On day-1 prior to transplantation, recipient chimeric mice (B6/BA or IL-12p40 KO) (CD45.2, CD90.2) received a total of 9 Gy irradiation, divided into two doses of 4.5 Gy given 3 hours apart [39]. On day 0, mice were injected i.v. with 5 × 10^6^ T-cell depleted (TCD) BM cells from FVB donors (H-2K^q^ CD45.1, CD90.1) along with 3 × 10^5^ MACS-purified T-cells from *luc +* FVB donors. FVB mice that had not undergone an initial radiation chimera transplant were used as syngeneic control mice, and received a transplant identical to the radiation-chimera mice. Secondary transplants corresponding to experiments shown in Figures 4 and 5 were done using BA.B10 (H2K^k^, CD45.2, CD90.1) bone marrow donors and B10.BR (H2K^k^, CD45.2, CD90.2) T-cell donors. All transplant recipients were monitored for survival and clinical signs of GvHD daily. Mice were scored for clinical signs of GvHD by weight loss, posture, activity, fur texture, and skin integrity twice weekly for the 30 days, and then weekly as described by Cooke et al. [13]. According to IACUC protocol, moribund mice, mice with greater than 25% weight loss, and mice surviving until the end of the experiment, were euthanized and considered to have died on the day following euthanasia for analysis of post-transplantation survival.

### Flow cytometry

For chimerism analysis, peripheral blood was collected on days 30, 60 and 100 (+/- 5 days) in tubes containing 20 μl heparin from the tail vein of transplant recipients. For flow cytometric analysis, red blood cells were lysed via incubation in an ammonium chloride lysis buffer. Host- and donor-derived leukocytes and T-cells were measured by flow cytometry using monoclonal antibodies for specific leukocyte markers expressed on B6, congenic B6 Pepboy, IL-12p40 KO, FVB, B10.BR, and BA.B10 strains (anti-mouse CD3-APC, CD45.1-APC-Cy7, CD45.2-FITC, H2k^b^-PE; BD Biosciences, San Diego, CA). Spleen samples were obtained on day 10 post transplant and intracellular cytokine expression of IFN-γ, TNF-α, IL-10, and IL-17 by donor CD4+ and CD8+ T-cells was analyzed by using a Cytofix/Cytoperm Kit (BD Biosciences, San Diego, CA). Presence of donor regulatory T cells in the spleen on day 10 post transplant was analyzed by surface staining using anti-mouse CD3-PE-Cy7, CD90.2-APC, H-2K^k^-FITC, CD4-Alexa-700 and CD25-APC-Cy7, followed by intracellular staining of FoxP3-PE (BD Biosciences, San Diego, CA). All samples were analyzed on a FACS Canto (Beckton Dickinson, San Jose, CA) and list mode files were analyzed using FlowJo software (Tree Star, Inc 2007, Ashland, OR). Cells staining positive for cytokines and FoxP3 were determined as those exhibiting a fluorescence signal greater than that of a corresponding isotype-control antibody (BD-Biosciences).

### In vivo bioluminescent imaging

*In vivo* bioluminescent imaging (BLI) was performed using a cooled charge-coupled device (CCD) camera system (IVIS Imaging System 100, Xenogen, Alameda, CA, USA). Mice received a subcutaneous injection of D-luciferin (0.15 mg/g body weight; Firefly luciferin potassium salt, Xenogen, Alameda, CA, USA). Ten minutes later, mice were placed in the chamber of the CCD camera system under rodent cocktail (ketamine 20 mg/ml and xylazine 2.5 mg/ml, 0.1 ml/25 g body weight) anesthesia. Photographic and luminescent images in the ventral projection were obtained using a 3-minute exposure time. Up to five mice were imaged simultaneously. On each *in vivo* BLI image, a region of interest (ROI) encompassing the entire mouse was created, and the whole-body signal in the ROI (photons/sec) was quantified using Living Image software (Version 3.2, Xenogen, Alameda, CA, USA). The whole-body bioluminescent signal was used as a marker of the donor T-cell engraftment.

### Statistical analyses

Analyses of data were performed using Prism version 5 for Mac (GraphPad Software, San Diego, CA, http://www.graphpad.com). Data are presented as mean ± standard deviation (SD) of all evaluable samples. Survival differences between groups were calculated with the Kaplan-Meier log-rank test in a pair-wise fashion. Differences in the numbers of cells present in blood, bioluminescence ROI values, percentages of FoxP3+, cytokine-producing, and PD-1 or ICOS expressing cells were performed using a two-tailed unpaired Student’s t-test or a one-way analysis of variance (ANOVA) followed by a Bonferroni post-test to adjust for multiple comparisons. P-values of less than 0.05 were considered significant.

## Abbreviations

BM: Bone marrow; BMT: Bone marrow transplant; HSCT: Hematopoietic stem cell transplantation; HLA: Human leukocyte antigen; TCD: T cell depleted; TBI: Total body irradiation; GvL: Graft-versus-leukemia; GvHD: Graft-versus-host-disease; WT: Wild type; IL-12: Interleukin-12; KO: Knock out; B6: C57BL6/J; NK: Natural killer; DC: Dendritic cells; pDC: Plasmacytoid dendritic cells; mDC: Myeloid dendritic cells; MACS: Magnetic activated cell sorting; FACS: Fluorescence activated cell sorting; luc+: Luciferase-positive; i.v.: Intravenous; RBC: Red blood cell; WBC: White blood cell; PD-1: Programmed death-1; PDL1: Programmed death ligand-1; ICOS: Induced costimulator; TH1: T-helper-type-1; ROI: Region of interest; ANOVA: Analysis of variance; SD: Standard deviation.

## Competing interests

The authors declare that they have no competing interests.

## Authors’ contributions

KAD and YW designed and performed experiments, analyzed results and wrote the manuscript. JML, WACH, and CRG designed and performed experiments. CH performed experiments. EKW designed experiments, analyzed results and wrote the manuscript. All authors read and approved the final manuscript.
